# Convention of Superintendents of Hospitals for the Insane

**Published:** 1860-07

**Authors:** 


THE CONVENTION OF SUPERINTENDENTS OF HOSPITALS FOR THE INSANE was in session in this city during the last week of May. Thirty-two actual members were present, as well as a number by invitation, and many interesting papers on subjects appertaining to insane asylums were read.

